# Birthdate study of GABAergic neurons in the lumbar spinal cord of the glutamic acid decarboxylase 67-green fluorescent protein knock-in mouse

**DOI:** 10.3389/fnana.2013.00042

**Published:** 2013-12-09

**Authors:** Jing Huang, Jing Chen, Wen Wang, Yan-Yan Wei, Guo-Hong Cai, Nobuaki Tamamaki, Yun-Qing Li, Sheng-Xi Wu

**Affiliations:** ^1^Department of Anatomy, Histology and Embryology, K. K. Leung Brain Research Centre, Fourth Military Medical UniversityXi’an, China; ^2^Department of Morphological Neural Science, Graduate School of Medical Sciences, Kumamoto UniversityKumamoto, Japan

**Keywords:** GABAergic neuron, BrdU, birthdate, spinal cord, mouse

## Abstract

Despite the abundance of studies on γ-aminobutyric acid (GABA) ergic neuron distribution in the mouse developing spinal cord, no investigation has been devoted so far to their birthdates. In order to determine the spinal neurogenesis of a specific phenotype, the GABAergic neurons in the spinal cord, we injected bromodeoxyuridine (BrdU) at different developmental stages of the glutamic acid decarboxylase (GAD)_67_-green fluorescent protein (GFP) knock-in mice. We thus used GFP to mark GABAergic neurons and labeled newly born cells with the S-phase marker BrdU at different embryonic stages. Distribution of GABAergic neurons labeled with BrdU was then studied in spinal cord sections of 60-day-old mice. Our birthdating studies revealed that GABAergic neurogenesis was present at embryonic day 10.5 (E10.5). Since then, the generation of GABAergic neurons significantly increased, and reached a peak at E11.5. Two waves for the co-localization of GABA and BrdU in the spinal cord were seen at E11.5 and E13.5 in the present study. The vast majority of GABAergic neurons were generated before E14.5. Thereafter, GABA-positive neuron generation decreased drastically. The present results showed that the birthdates of GABAergic neurons in each lamina were different. The peaks of GABAergic neurogenesis in lamina II were at E11.5 and E13.5, while in lamina I and III, they were at E13.5 and E12.5, respectively. The present results suggest that the birthdates of GABAergic neurons vary in different lamina and follow a specific temporal sequence. This will provide valuable information for future functional studies.

## INTRODUCTION

Neurons of the dorsal spinal cord relay sensory information, while the ventral spinal cord contains motor neurons and interneurons that coordinate motor output. To establish the correct circuitry, different types of neurons in the spinal cord are formed in a defined spatial and temporal order during development (Caspary and Anderson, 2003; Helms and Johnson, 2003). It is well known that γ-aminobutyric acid (GABA) is a major inhibitory neurotransmitter in the central nervous system, and its physiological roles are well documented in both dorsal and ventral horn of the spinal cord (Schneider and Fyffe, 1992; Malcangio and Bowery, 1996). Previous investigations have revealed that early in development, GABA acts as an excitatory neurotransmitter and a trophic factor to influence growth, synapse maturation, and cell death (Owens and Kriegstein, 2002a,b; Herlenius and Lagercrantz, 2004), while later in development, it switches from excitatory to inhibitory and leads to mature functional neural networks (Branchereau et al., 2002; Stein et al., 2004; Rivera et al., 2005). GABAergic neurons have been postulated to be a critical component of local circuits in the adult spinal cord, which may influence the primary afferent terminals, projection neurons, other interneurons, and terminals of the descending inhibitory system in the superficial laminae (Millan, 2002). To better understand the role of GABAergic neurons in spinal cord, the expression pattern and time course of generation of the major cell types need to be established. These studies will help define and interpret patterns of gene expression, waves of differentiation, timing and extent of competence, and many other processes involved.

Indeed, the spatial and temporal development of GABA immunoreactivity has been studied in the embryonic rat (Ma et al., 1992; Tran and Phelps, 2000; Tran et al., 2003) and mouse spinal cord (Allain et al., 2004; Huang et al., 2007) both *in vivo* and *in vitro*. Antiserums to GABA (Todd and McKenzie, 1989; Todd and Sullivan, 1990; Proudlock et al., 1993) or its synthetic enzyme glutamic acid decarboxylase (GAD; McLaughlin et al., 1975; Tillakaratne et al., 2000; Mackie et al., 2003) have been used for immunohistochemical analysis. By using the GAD_67_-GFP knock-in mouse, we previously observed the expression pattern of GABAergic neurons in the developing spinal cord (Huang et al., 2007). The results revealed that the GABAergic population followed a rostro-caudal and ventro-dorsal gradient of maturation. The birthdates of GABA-immunoreactive neurons have been described in different areas of the nervous system, such as the developing rat dentate gyrus (Dupuy and Houser, 1997), rat retina (Lee et al., 1999) and developing mouse visual cortex (Yozu et al., 2004). However, birthdate dependent layer positioning of GABAergic neurons in the mouse spinal cord has not yet been fully understood.

In order to determine the spinal neurogenesis of GABAergic cells in the spinal cord, we labeled newborn cells with S-phase marker bromodeoxyuridine (BrdU) on designated embryonic days. The GAD_67_-GFP knock-in mice were used, in which a GFP gene was introduced into the gene for GAD_67_ and all GABAergic neurons were fluorescent (Tamamaki et al., 2003). BrdU was injected at different stages during development and spinal sections were labeled for GABA and BrdU at 60 days after birth. This strategy enabled us to obtain data regarding the generation timetable, migration profile, and final position of GABAergic neurons incorporating BrdU during their last mitosis.

## MATERIALS AND METHODS

### EXPERIMENTAL ANIMALS

The generation and characterization of the GAD_67_-GFP knock-in mouse have been described previously (Tamamaki et al., 2003). Twenty-six pregnant GAD_67_-GFP knock-in mice were used in the present study. However, the offsprings of two pregnant mice died. So, the 24 remaining litters consisted of eight groups of three litters, each group injected with BrdU at a different developmental age. Each litter comprised four to six animals included in this study (see **Table 1**). The day when the vaginal plug was found was considered embryonic day 0 (E0), and birth usually occurred at E19, which was designated as postnatal day 0 (P0). GAD_67_-GFP knock-in fetuses could easily be distinguished from the wild type under the detection of fluorescence at the microscope. Experimental mice were housed and treated in strict accordance with the Rules for Animal Care and Use for Research and Education of Fourth Military Medical University.

**Table 1:** Time and animal numbers designed for BrdU injections.

Age	Animals analyzed (litters)
E10.5	6 (3)
E11.5	6 (3)
E12.5	6 (3)
E13.5	6 (3)
E14.5	6 (3)
E15.5	6 (3)
E16.5	6 (3)
E17.5	4 (3)

*The table lists the number of mice analyzed for each given injection time point. The number of independent litters from which animals were derived is indicated in parentheses.*

### BrdU INJECTION

Timed-pregnant mice were given an intraperitoneal injection of BrdU (Sigma, St. Louis, MO, USA; 50 mg/kg body weight), a marker of cell proliferation, at E10.5, E11.5, E12.5, E13.5, E14.5, E15.5, E16.5, and E17.5. The injection protocol used was a modified version of those described previously (Miller and Nowakowski, 1988; Takahashi and Wakita, 1993). Offspring for these studies were anesthetized and prepared for morphological study at P60.

### TISSUE PREPARATION

Pups were terminally anesthetized 60 days after birth and perfused intracardially with approximately 50 ml of 0.1 M phosphate buffer (PB, pH 7.4) containing 4% paraformaldehyde. Lumbar segments 3–5 of the spinal cord were obtained and post-fixed in the same fixative for 4 h at room temperature (RT), placed in 0.1 M PB containing 30% (w/w) sucrose overnight at 4°C. Coronal spinal sections (30 μm thickness) were prepared in a cryostat, and free-floating sections were stored at 4°C in 0.01 M phosphate buffered solution (PBS, pH 7.4) until further processing.

### BrdU AND GFP DOUBLE IMMUNOFLUORESCENT STAINING

Sections were rinsed in 0.01 M PBS for three times (5 min each), blocked with 2% goat serum in 0.01 M PBS containing 0.3% (v/v) Triton X-100 at RT for 1 h, and then used for the double immunofluorescent staining. Sections were first incubated at RT overnight with rabbit anti-GFP antibody (1:1000, Sigma, USA) and further incubated at RT for 3 h with biotinylated donkey anti-rabbit IgG antibody (1:200, Chemicon, Temecula, CA, USA); the incubation medium was prepared by using 0.05 M PBS containing 0.3% (v/v) Triton X-100, 0.25% (w/v) λ-carrageenan, 5% (v/v) normal donkey serum, and 0.05% (w/v) NaN_3_ (PBS-XCD). The sections were then rinsed for 15 min in PBS and fixed with 4% paraformaldehyde for 10 min. For immunostaining of BrdU, sections were washed with PBS and denatured with 2 N HCl (30 min at 37°C). After neutralizing the acid by immersing slides in 0.1 M borate buffer (pH 8.0) for 5 min, sections were incubated at RT overnight with 1 μg/ml rat anti-BrdU antibody (Abcam, Cambridge, UK) and further incubated at RT for 3 h with 4 μg/ml Alexa Fluor 594-conjugated donkey anti-rat IgG (Molecular Probes, Eugene, OR, USA) and 1 μg/ml fluorescein isothiocyanate (FITC)-conjugated streptavidin (Molecular Probes). The sections were mounted onto gelatinized glass slides and cover slipped with a mixture of 50% (v/v) glycerol and 2.5% (w/v) triethylenediamine (anti fading reagent) in PBS. The specificities of the staining were tested on the sections in another dish by omission of the primary specific antibodies. No immunoreactive products were found on the sections as predicted.

### IMAGE ANALYSIS AND QUANTIFICATION

From each animal, a single section from lumbar segments 3–5 of the spinal cord was selected randomly and scanned with a confocal laser microscope (FV-1000, Olympus, Tokyo, Japan). In two of the mice from the E17.5 injected group (**Table 1**), two sections were selected instead. Sections at equivalent levels were stained using cresyl violet to reveal the general cytoarchitecture of the spinal cord. Borders between spinal cord layers were determined mainly by reference to Allen mouse spinal cord atlas and information obtained from cresyl violet staining. A cell was considered birthdated if the immunoreaction product filled more than half the area of the nucleus. For double immunofluorescence labeling results, the total number of positive neurons in the spinal cord was counted respectively from six sections randomly selected from four to six animals. These animals were from three different litters. The relative quantitative analysis of BrdU and GFP double-labeled neurons was performed by counting the number of double-labeled somata on each section. Profile counts were corrected by using Abercrombie’s formula. For each frame, the thickness of the section was measured by focusing on the upper and lower section surfaces. The longest diameter of cell body through the nucleus was regarded as the major diameter of the cell. We randomly selected 50 cells, measured the major diameter of the nuclei, and calculated the mean nuclear diameter. Then, the numbers of counted cells were corrected by use of Abercrombie’s equation: number of cell = number of cells counted × *T*/(*T* + *h*), where *T* = thickness of the sections and *h* = the mean diameter of the nuclei of the cells (Abercrombie, 1946; Guillery, 2002).

Statistical differences were assessed by non-parametric method, namely Kruskal–Wallis test. We presented each data point for each embryonic age, plus the median as a horizontal trace. Data are presented as median with interquartile range. The statistical analysis was performed using SPSS software for Windows, version 16.0 (SPSS Inc. Chicago, IL, USA). For all analyses, *P* < 0.05 was considered statistically significant.

All images were processed and adjusted for brightness and contrast using Adobe Photoshop 7.0 (Adobe Systems Inc, Mountain View, CA, USA).

## RESULTS

**FIGURE 1:**
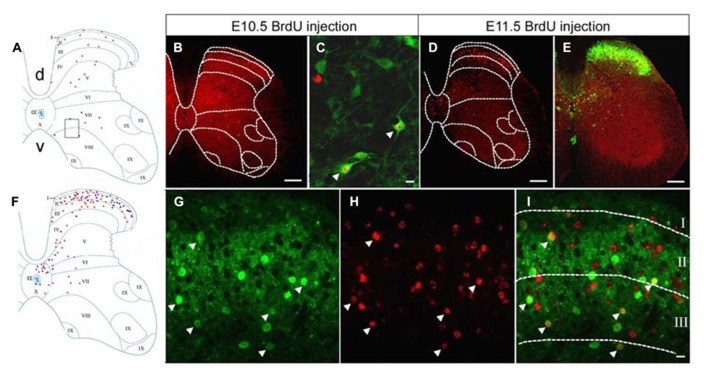
**Double-labeling of GFP (green) and BrdU (red) in the spinal cord of the GAD_67_-GFP knock-in mice following injection of BrdU at E10.5 and E11.5.** **(A,F)** Schematic drawings showing the distribution of BrdU-positive cells and double-labeled neurons in the lumbar spinal cord, taken from P60 mice that had received a single pulse of BrdU at E10.5 **(A)** and E11.5 **(F)**. **(A)** The schematic drawing represents a single section and it corresponds to the same section illustrated in **(B)**. **(F)** The schematic drawing represents a single section and it corresponds to the same section illustrated in **(D)**. Red circles correspond to BrdU-positive cells and blue circles correspond to double-labeled neurons. **(B)** Note the distribution of BrdU-positive cells after injection of BrdU at E10.5. **(C)** Higher magnification obtained from a different section, in the approximate position indicated by the rectangle in **(A)**. Double-labeled cells for GFP and BrdU were distributed in the ventral horn (arrowheads). **(D)** Distribution of E11.5-birthdated BrdU-positive nuclei in the spinal cord. **(E,G–I)** Double-labeled cells for GFP and BrdU were highly concentrated in the spinal dorsal horn after injection of BrdU at E11.5. Double-labeled cells are indicated by white arrowheads. The borders of the spinal cord lamina are depicted as dashed lines. In **(A)**, “d” is dorsal; “v,” ventral; and “cc” indicates the central canal. The same section orientation is maintained in the following figures. Scale bars = 100 μm in **(B,D,E)** and 10 μm in **(B,I)** (applies to **G–I**).

The co-localization of GFP and BrdU was examined within neurons of the lumbar spinal cord taken from P60 GAD_67_-GFP knock-in mice, which had been injected with BrdU at different embryonic stages. BrdU-positive cells were clearly classifiable as either GFP-positive or -negative populations. Double-labeled cells for GFP and BrdU represented GABAergic neurons that were undergoing DNA synthesis during the last cell cycle when BrdU was injected. These double-labeled cells were easily recognized by the large green cell body surrounding a BrdU-labeled red nucleus (**Figure 1**). Cells labeled with BrdU were sparsely distributed in the gray matter of the spinal cord after injections of BrdU at E10.5 (**Figures 1A,B**). The double-labeled GFP/BrdU cells were mainly located in the ventral horn and in the intermediate zone (**Figures 1A,C**). The number of double-labeled cells was exiguous, suggesting that GABAergic neurogenesis was certainly minor at E10.5.

After injections of BrdU at E11.5, BrdU-labeled cells were spread widely throughout the spinal cord. Most of the BrdU-labeled cells were found in the superficial dorsal horn, the medial part of the deep dorsal horn, and in the area around the central canal (**Figures 1D,F**), which was a pattern of GABAergic neuronal distribution similar to the one found in the adult GAD_67_-GFP knock-in mouse (**Figure 1E**). Intense double-immunoreactivity was observed in the superficial layer of the spinal cord and in the area around the central canal (**Figures 1E–I**).

**FIGURE 2:**
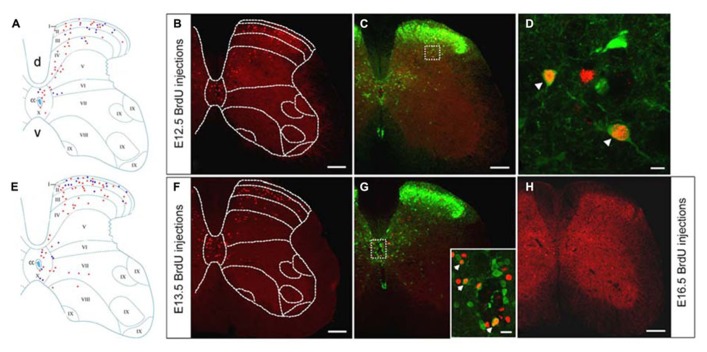
**Double-labeling of GFP (green) and BrdU (red) in the spinal cord of the GAD_67_-GFP knock-in mice following injection of BrdU at E12.5 and E13.5.** **(A,E)** Schematic drawings showing the distribution of BrdU-positive cells and double-labeled neurons in the lumbar spinal cord following injection of BrdU at E12.5 **(A)** and E13.5 **(E)**. **(A)** The schematic drawing represents a single section and it corresponds to the same section illustrated in **(B)**. **(E)** The schematic drawing represents a single section and it corresponds to the same section illustrated in **(F)**. Red circles correspond to BrdU-positive cells and blue circles correspond to double-labeled neurons. **(B,C)** Distribution of the BrdU-labeled cells and double-labeled neurons in the spinal cord after injection of BrdU at E12.5. **(D)** Magnified image of the rectangle indicated in **(C)**. Note the higher magnification of double-labeled cells for GFP and BrdU in the deep dorsal horn. **(F)** The distribution of E13.5-birthdated BrdU-positive nuclei was mainly located in the spinal dorsal horn. **(G)** The inset in **(G)** illustrates double-labeled cells for GFP and BrdU in the area around the central canal. **(H)** Distribution of BrdU-positive cells in the spinal cord after injection of BrdU at E16.5. Double-labeled cells are indicated by white arrowheads **(D,G)**. The borders of the spinal cord lamina are depicted as dashed lines. Scale bars = 100 μm in **(B,C,F,H)** (apply to **G**). Scale bars = 10 μm in **(D)** and 20 μm in the inset in **(G)**.

BrdU-labeled cells were arranged prominently in the spinal dorsal horn after injections of BrdU at E12.5 (**Figures 2A,B**). More GFP^+^/BrdU^+^ cells were distributed in the deep dorsal horn, as compared with the small number of double-labeled cells remaining in the superficial layer of the spinal cord (**Figures 2C,D**). These results indicated that the GABAergic neurons generated at E12.5 were mainly present in the deep dorsal horn.

After injections of BrdU at E13.5, BrdU-labeled cells were scattered throughout most of the spinal cord, but tended to be more common in the dorsal horn and the area around the central canal (**Figures 2E,F**). Double-labeled cells were found in the dorsal horn as well as in the area around the central canal (**Figures 2E,G**), suggesting that part of GABAergic neurons of the superficial spinal cord were generated at E13.5.

**FIGURE 3:**
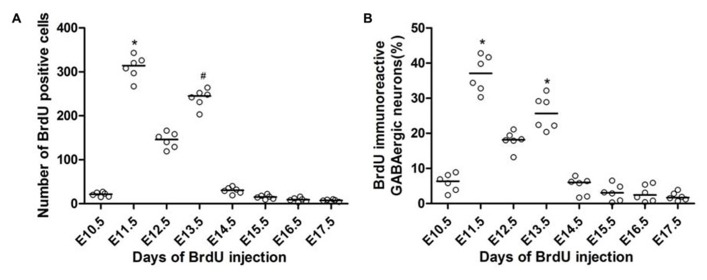
**Quantitative analysis of BrdU-positive cells (A) and the co-localization of GFP and BrdU **(B)** in the spinal cord of the P60 GAD_**67**_-GFP knock-in mice following injections of BrdU at different developmental stages.** The *x*-axis depicts the embryonic stages in which BrdU was injected, and the *y*-axis depicts the number of BrdU-positive cells or the percentage of double-labeled cells among all GABAergic neurons, respectively. The horizontal bar indicates the median. Statistical differences were assessed by non-parametric method, namely Kruskal–Wallis test. *P < 0.05 compared with that of E10.5, E14.5, E15.5, E16.5, and E17.5. ^#^*P* < 0.05 compared with that of E10.5, E15.5, E16.5, and E17.5.

After injection of BrdU at E14.5, only few BrdU-labeled cells and GFP^+^/BrdU^+^ cells could be detected within the lumbar spinal cord (**Figure 3**). This result indicated that most neurons were born at or before E14.5 and that a very small fraction of GABAergic neurons was generated after E14.5. Cells labeled after E16.5, in addition to being few in number (**Figure 3**), were also scattered throughout the gray matter of the spinal cord (**Figure 2H**).

This birthdate study revealed that the overall production of GABAergic neurons was mainly limited during early neurogenesis (E10.5–E14.5), especially during E11.5 and E13.5 (**Figure 3B**). A few GABAergic neurons were labeled with BrdU as early as E10.5 (**Figure 3B**). The number of GFP^+^/BrdU^+^ cells following BrdU injection at E11.5 was remarkably increased compared to that at E10.5, accounting for nearly 37.1 (32.18–41.98)% of the overall GFP-positive neurons. Neurogenesis reached peaks at E11.5 and E13.5, and then rapidly tapered off at E14.5, with very little neurogenesis occurring after E16.5 (**Figure 3**).

**FIGURE 4:**
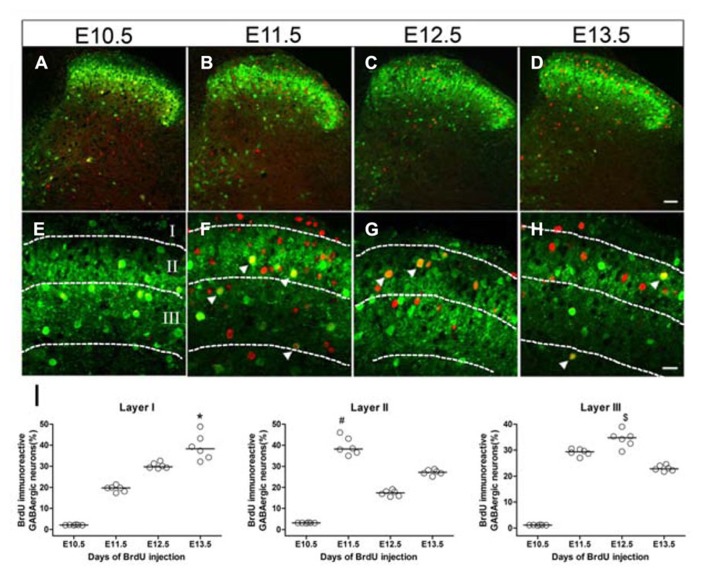
**Laminar distribution of double-labeled cells for GFP and BrdU in the superficial dorsal horn of GAD_67_-GFP knock-in mice injected with BrdU at E10.5 **(A,E)**, E11.5 **(B,F)**, E12.5 **(C,G)**, and E13.5 **(D,H)**, respectively.** Double-labeled cells are indicated by white arrowheads **(E–H)**. **(I)** Data showing the percentage of double-labeled cells among GABAergic neurons in lamina I, lamina II, and lamina III of spinal cord at different developmental stages. The horizontal bar indicates the median. Statistical differences were assessed by non-parametric method, namely Kruskal–Wallis test. *P < 0.05 compared with that of E10.5 and E11.5. ^#^*P* < 0.05 compared with that of E10.5 and E12.5. ^$^*P* < 0.05 compared with that of E10.5 and E13.5. Borders of lamina I, lamina II, and lamina III are depicted as dashed lines. Scale bars = 50 μm in **(D)** (applies to **A–D**) and 20 μm in **(H)** (applies to **E–H**).

Since GFP fluorescence was highly concentrated in the superficial dorsal horn of the GAD_67_-GFP knock-in mouse, we focused on the neurogenesis of GABAergic neurons in laminae I–III of the spinal cord. **Figure 4** shows the GFP and BrdU double-labeled cells in spinal cords of mice injected with BrdU at different embryonic stages. The peaks of GABAergic neurogenesis in lamina II were at E11.5 and E13.5 (**Figures 4B,D,F,H,I**), while in lamina III, the majority of the GABAergic neurons were produced at E12.5 (**Figures 4C,G,I**), and in lamina I, the peak was at E13.5 (**Figures 4D,H,I**). These results indicated that laminar distribution of the GABAergic neurons may be related to the neurogenesis stage.

## DISCUSSION

In the present study, the generation timetable of GABAergic neurons during certain developmental periods in the lumbar spinal cord was investigated by using the GAD_67_-GFP knock-in mouse. Our results clearly demonstrated that birthdates of GABAergic neurons vary and follow a specific temporal sequence.

Previous studies showed that neurons in the mouse spinal cord are largely born between E10 to E14 (Nornes and Carry, 1978; Caspary and Anderson, 2003; Helms and Johnson, 2003). The neurons of the dorsal horn originated from E12 to E14, while the ones in the intermediate gray region originated from E11 to E14, as well as the motor neurons in the ventral horn originated on E10 and E11 (Nornes and Carry, 1978). Consistent with previous studies, the present study demonstrated that the overall neurogenesis in the lumbar spinal cord was mainly limited to the interval between E10.5 and E14.5, and the labeling pattern of BrdU varied according to the developmental stages. Although these analyses provided an overview of the time and sites of origin, the detailed generation timetable of the GABAergic neurons in the spinal cord is still unknown.

Our birthdates study showed that the vast majority of GABAergic neurons were generated before E14.5 and the birthdates of GABAergic neurons in each lamina were different. In mice, neurons in the dorsal horn are generated in two phases: an early phase (E10–E11.5) that generates the relay neurons and sensory interneurons of the deep dorsal horn, followed by a second phase (E12–E13.5), in which most of the association interneurons that form the superficial dorsal horn are born (Caspary and Anderson, 2003; Helms and Johnson, 2003; Chizhikov and Millen, 2005). In previous studies, two early-born cell types, dI4 and dI5 neurons, generate sensory interneurons that predominantly settle in the deep dorsal horn, while the late-born neurons (dILA and dILB) migrate along a dorsolateral direction and form the superficial dorsal horn (Altman and Bayer, 1984; Gross et al., 2002; Muller et al., 2002; Caspary and Anderson, 2003; Helms and Johnson, 2003). dI4 and dILA neurons differentiate as GABAergic neurons that express the HD transcription factors Pax2 and Lhx1/5, whereas dI5 and dILB neurons develop as glutamatergic neurons that express Tlx1/3 and Lmx1b (Gross et al., 2002; Cheng et al., 2004; Mizuguchi et al., 2006). Our results about the birthdates and distribution of GABAergic neurons are similar to these reports, although there are some discrepancies. Previous studies showed that interneurons in the superficial dorsal horn were born in the second phase (E12–E13.5; Gross et al., 2002; Muller et al., 2002; Helms and Johnson, 2003), while our results showed that many GABAergic neurons in the superficial dorsal horn were generated at E11.5. The above-mentioned studies identified the GABAergic neurons based on the HD transcription factors, while we used the GFP antibody to identify the GABAergic neurons in the GAD_67_-GFP knock-in mouse. This difference may account for the discrepancies with previous studies regarding the birthdates and distribution of GABAergic neurons.

When comparing the neurogenesis pattern of GABAergic neurons and that of the total neuronal population in the spinal cord, we could see that there are two peaks during neurogenesis, one at E11.5 and another at E13.5. This could be further confirmed by the birthdate of GABAergic neurons in laminae I–III. The present findings show that there are important differences in the timing of neurogenesis in individual layers in the spinal cord, which may reflect a general trend of the nervous system development. It has been reported that the ratio of co-localization of GABA and BrdU showed a peak value on E18 in the central rat retina and on E20 in the periphery retina (Lee et al., 1999). In the developing mouse visual cortex, GABAergic neurons born on E12.5 were distributed around two peak locations, mainly around layer V and layer II/III, while the cells born on E15.5 exhibited only one peak distribution in layer II/III (Yozu et al., 2004). This phenomenon may be attributed to the different birthdates and distribution of the subpopulation of GABAergic neurons in the spinal cord, such as, the subtypes of Ca^2^^+^ binding proteins and neuropeptides (Tamamaki et al., 2003; Heinke et al., 2004).

In summary, the present study demonstrates that the overall generation of GABAergic neurons was mainly limited to E10.5–E14.5 in the mouse lumbar spinal cord. The laminar distribution of the GABAergic neurons was related to the developmental stage. A previous study showed that the mouse embryonic GABAergic population in the cervical and lumbar spinal cord matures with similar pattern following a rostro-caudal gradient (Allain et al., 2004). Although additional studies are still required, we propose that the birthdates of GABAergic neurons in other spinal cord segments have a developmental pattern similar to that of the lumbar spinal cord.

## Conflict of Interest Statement

The authors declare that the research was conducted in the absence of any commercial or financial relationships that could be construed as a potential conflict of interest.
